# Observation of lithium stripping in super-concentrated electrolyte at potentials lower than regular Li stripping†

**DOI:** 10.1039/d1ra01490k

**Published:** 2021-04-09

**Authors:** Tohru Shiga, Yumi Masuoka, Hiroshi Nozaki

**Affiliations:** Toyota Central Research & Development Laboratories Inc. Yokomichi Nagakute-city Aichi-ken 480-1192 Japan e0560@mosk.tytlabs.co.jp

## Abstract

Lithium plating/stripping was investigated under constant current mode using a copper powder electrode in a super-concentrated electrolyte of lithium bis(fluorosulfonyl)amide (LiFSA) with methylphenylamino-di(trifluoroethyl) phosphate (PNMePh) and vinylene carbonate (VC) as additives. Typical Li plating/stripping for Cu electrodes in organic electrolytes of conventional lithium batteries proceeds at potentials of several millivolts *versus* a Li counter electrode. In contrast, a large overpotential of hundreds of millivolts was observed for Li plating/stripping with the super-concentrated electrolyte. When Li stripping started immediately after Li plating and with no rest time between plating and stripping, two potential plateaus, *i.e.*, two-step Li stripping, was observed. The potential plateau for the 1^st^ stripping step appeared at −0.2 V *versus* a Li metal counter electrode. The electrical capacity for the 1^st^ stripping step was 0.04 mA h cm^−2^, which indicates irregular Li stripping. Two-step Li stripping was also recorded using cyclic voltammetry. The electrochemical impedance spectroscopy (EIS) studies indicated that the two-step Li stripping behaviour reflected two different solid electrolyte interphases (SEIs) on electrodeposited Li in a Cu electrode. The SEI for the 1^st^-step stripping was in a transition period of the SEI formation. The open circuit voltage (OCV) relaxation with an order of tens of hours was detected after Li plating and before Li stripping. The *in operando* EIS study suggested a decrease of the charge transfer resistance in the Cu powder electrode during the OCV relaxation. Since the capacitance for the voltage relaxation was a dozen microfarads, it had a slight contribution to the 1^st^-step Li stripping behaviour. The voltage relaxation indicated the possibility that it is difficult for Li ions to be electrodeposited or that the Li plating is in a quasi-stable state.

## Abstract 2

When Li plating/stripping was conducted on a Cu powder electrode in a super-concentrated electrolyte of LiFSA and PNMePh, two potential plateaus of the Cu electrode for Li stripping were observed at −0.2 V and +1.0 V *versus* a Li counter electrode.

## Graphical abstract

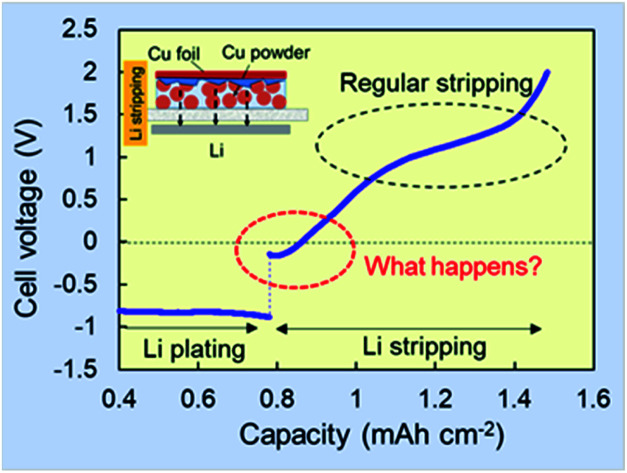

## Introduction

There have been recent requests that conventional lithium-ion batteries have increased energy capabilities toward application in future electric vehicles. The use of Li metal as the anode is one approach to this challenge because of its high theoretical specific capacity (3862 mA h g^−1^) and low operation potential (−3.04 V *versus* the standard hydrogen electrode). However, there is a serious problem that compromises battery safety, *i.e.*, the growth of dendritic Li.^1,2^ Lithium dendrites lead to the short-circuiting of a cell, which produces fumes and combustion of the organic electrolyte. To address this issue, the morphology of Li plating has been visualized by *in situ* methods such as atomic force microscopy,^3^ nuclear magnetic resonance spectroscopy (NMR),^4,5^ and transmission X-ray microscopy.^6^ Many techniques to suppress the growth of Li dendrites have also been proposed, such as electrolyte modification,^7,8^ and surface coating.^9–13^ The mechanism of dendrite growth must also be elucidated fundamentally. Based on previous studies on the mechanism of Li dendrite growth,^14–17^ it has been accepted that the formation of Li dendrites can be explained by the following multiple stages: mass transfer of Li^+^ in the electrolyte, mass transfer of Li^+^ in the solid electrolyte interphase (SEI), interface charge transfer, surface movement and electrochemical nucleation of Li atoms, growth of Li nuclei, and dendrite growth. A deep understanding of the fundamentals of Li plating/stripping behaviour is thus important to suppress Li dendrite growth. Super-concentrated electrolytes have recently attracted much attention as a new research field for physical chemistry.^18–21^ Among several researches on the suppression of dendritic Li growth with super-concentrated electrolyte systems, Zhang and colleagues reported a high concentration of an imide-type supporting salt for high rate and stable cycling performance.^22^ A new solvent-in salt electrolyte for the suppression of metallic Li dendrite growth has been proposed by the team of Beijing National Laboratory.^23^ We have previously reported the battery performance of graphite/Li half cells using super-concentrated electrolytes with self-extinguishing solvents, such as fluorinated alkyl phosphates and fluorinated phosphoric ester amide,^24,25^ based on the high flammability risk that accompanies organic solvents. More recently, we have examined electrochemical interaction between Li ion and cerium dioxide^26^ or yttrium oxide^27^ in a super-concentrated electrolyte composed of lithium bis(fluorosulfonyl)amide (LiFSA) with methylphenylamino-di(trifluoroethyl) phosphate (PNMePh). Here, Li plating/stripping was investigated using a Cu powder electrode and a super-concentrated electrolyte composed of LiFSA with PNMePh and vinylene carbonate (VC) as an electrolyte additive. We report new electrochemical behaviour, *i.e.*, two-step Li stripping in the super-concentrated electrolyte.

## Experimental

### Materials

Copper (Cu) powder with an average particle diameter of 1 μm was obtained from Kojundo Chemical. LiFSA (Fig. S1†) and VC (battery grade) an electrolyte additive^28,29^ were purchased from Kishida Chemicals. LiFSA was dried at 150 °C under vacuum for 5 h before use. PNMePh (Fig. S1†) with a water content of 37 ppm was obtained from Tohso Finechem Corporation.

### Electrochemical measurements

The Cu powder electrode was prepared as follows. The Cu powder (98% by weight) was mixed with polyvinylidene difluoride (2% by weight, #9350, Kureha) and *N*-methylpyrrolidone (NMP; Wako Chemicals) using a kneading machine (ARE-310, Thinky Co., Ltd) at 2200 rpm for 5 min. The electrode slurry was spread onto a copper current collector (20 μm thick) using a doctor-blade technique and dried at 150 °C under vacuum for 5 h. The electrode sheet was pressed to obtain a 14 mm diameter disk electrode with a thickness of 55 μm (Fig. S2†). The loading of Cu powder was 28–31 mg cm^−2^. A half coin cell (Fig. S3†) was fabricated using 14 mm diameter disk electrodes, two filter papers (200 μm thick, Advantec, 5C), an 18 mm diameter and 0.4 mm thick Li metal disk (Honjo Metal), and the super-concentrated electrolyte. The super-concentrated electrolyte was prepared by mixing LiFSA and PNMePh in a molar ratio of [Li salt]/[solvent] = 0.333 and the diluted electrolyte was prepared with a molar ratio of [LiFSA]/[PNMePh] = 0.125. The concentration of VC added to the electrolyte was 1 vol%. The cells were fabricated using a dilute electrolyte with a molar ratio of [Li salt]/[solvent] = 0.125 in the same manner. The half coin cell was connected to a charge–discharge unit (Hokuto Denko, HJ1001SM8A). Charging (Li plating) was conducted at 0.05 mA (=0.033 mA cm^−2^) for 24 h, and discharging (Li stripping) was conducted up to 2.0 V with a constant current of 0.05 mA.

### Cyclic voltammetry (CV)

The half coin cell was connected to a potentiometer (IVIUM Technologies, IVIUMSTST-XR) to measure CV profiles for Li plating/stripping and Li intercalation in the diluted or highly concentrated electrolyte. The Li metal anode was used as both counter and reference electrodes. Cyclic voltammetry for the electrolytes was performed with sweep rates between 0.1 mV s^−1^ and 0.5 mV s^−1^ at 25 °C.

### Electrochemical impedance spectroscopy (EIS)

The Cu powder cathode in the coin cell was set as the working electrode, and the Li metal anode as counter and reference electrodes. EIS was recorded at 25 °C in the frequency range from 7 MHz to 20 mHz with a potentiostat (BioLogic, SP-300) to investigate the formation of a passive layer on the Cu electrode. The amplitude of the sinusoidal potential was adjusted by 10 mV. Impedance spectra were fitted using EC-Lab Zfit of the SP-300 potentiostat.

### Analysis

Electrodeposition of the Li in the sample was examined using field emission scanning electron microscopy (FE-SEM; JEOL, JSM-7000F). Energy-dispersive X-ray spectroscopy (EDX) was also performed to identify passivation films on Li. The identification of Li in the Cu electrode after the Li plating/stripping test was conducted using ^7^Li-NMR (Bruker, AVANCE 400). The measurement conditions are summarized in Table S1.† The viscosity of the electrolyte was measured under regular flow mode using a cone-and-plate type viscometer (Haake, RheoStress 600). The shear rates were between 10 s^−1^ and 10^3^ s^−1^

## Results and discussion

### Lithium plating/stripping

**Fig. 1:**
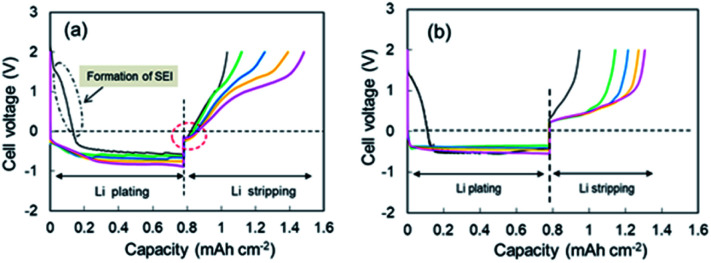
1^st^ through 5^th^ cycle voltage–capacity curves for cells with (a) the super-concentrated electrolyte and (b) the dilute electrolyte. Cycle number: 1^st^ (black), 2^nd^ (green), 3^rd^ (blue), 4^th^ (orange), and 5^th^ (pink).

Continuous deposition of Li to the Cu electrode was conducted at a current of 0.05 mA (0.033 mA cm^−2^). The 1^st^ through 5^th^ voltage–capacity curves of the cell using the super-concentrated electrolyte are shown in Fig. 1a. The horizontal axis represents the electrical capacity per area of Cu electrode. Li plating started at 2.6 V *vs.* Li^+^/Li. A large wave due to the reductive decomposition of VC and PNMePh, and subsequent SEI formation was observed at the initial stage of the 1^st^ cycle, of which the electrical capacity was *ca.* 0.15 mA h cm^−2^. A flat voltage was observed at −0.6 V, which reflects Li plating. The large overpotential is due to the high viscosity of the electrolyte. When the electric current was reversed at 0.78 mA h cm^−2^, the cell showed two-step Li stripping, *i.e.*, two voltage plateaus were observed at −0.2 V and +1.0 V. Fig. S4† shows an enlarged view of the Cu electrode voltage–capacity curve during Li stripping. The first negative voltage region with a capacity of 0.04 mA h cm^−2^ was detected in every cycle. In contrast, the cell with the dilute electrolyte ([LiFSA]/[PNMePh] = 0.125) showed no negative voltage region (Fig. 1b). The voltage–capacity curves for cells with the electrolytes using various LiFSA concentrations are summarized in Fig. S5.† The 1^st^ step Li stripping became unclear in the electrolyte with [LiFSA]/[PNMePh] = 0.25.

### Cyclic voltammetry

**Fig. 2:**
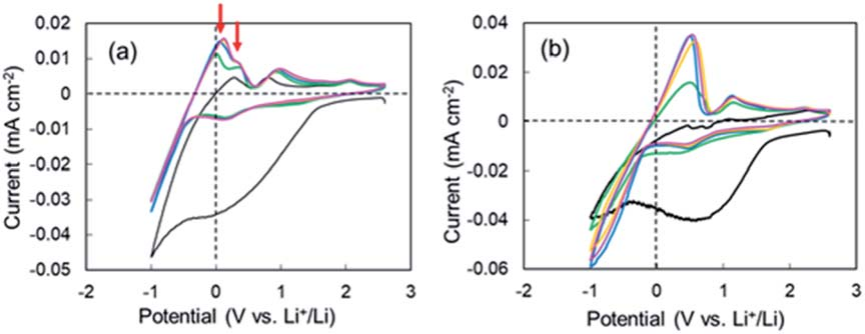
1^st^ through 5^th^ CV profiles of the cell with (a) the super-concentrated electrolyte and (b) the dilute electrolyte. Cycle number: 1^st^ (black), 2^nd^ (green), 3^rd^ (blue), 4^th^ (orange), and 5^th^ (pink).

CV measurements were performed to elucidate the negative voltage region in the super-concentrated electrolyte. CV profiles up to the 5^th^ cycle for the cell with the super-concentrated electrolyte are shown in Fig. 2a. The sweep rate was 0.2 mV s^−1^. In the 1^st^ cathodic scan, a large wave due to the reductive decomposition of VC and PNMePh, and subsequent SEI formation was observed between +1.5 V and −0.5 V. The cathodic current increased linearly below −0.5 V as the potential was decreased. This increase reflects Li plating. When the 1^st^ anodic scan started, one peak was observed between 0 V and +0.3 V. By contrast one peak and one shoulder appeared in the same potential range from 2^nd^ cycle to the 5^th^ cycle (red arrows in Fig. 2a). The former signal reflects the negative voltage region in Fig. 1a. As shown in Fig. 2b, the two signals were not detected in the diluted electrolyte. To evaluate the negative region of both behaviours, CV curves were also measured at a sweep rate of over 0.5 mV s^−1^; however, the signal near 0 V disappeared under these measurement conditions (Fig. S6†). The intensity of the signal between +1.5 V and −0.5 V in the cathodic scan diminished significantly in the 2^nd^ to 5^th^ cycles. The small cathodic waves at +0.14 V in the 2^nd^ to 5^th^ cycles indicate that a stable SEI was formed at the 1^st^ scan, which prevented further electrolyte decomposition. These signals corresponded to the anodic peaks at +0.94 V, which suggests a reversible protective surface layer. It is well known that an ethylene carbonate-containing electrolyte can form a stable SEI where some of the SEI slowly fractures and reforms.^30,31^ Therefore, it was reasonable that the super-concentrated electrolyte in this study also formed a reversible SEI.

### Li stripping behaviour in some super-concentrated electrolytes

**Fig. 3:**
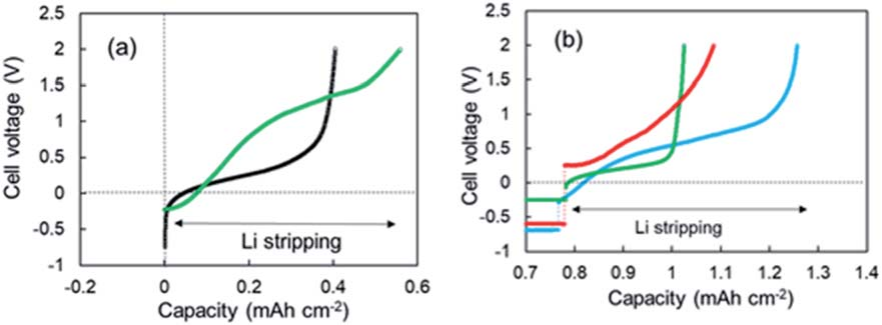
(a) 3^rd^ cycle voltage–capacity curves for the cells using the super-concentrated electrolytes with (green) and without (black) VC during the Li stripping process, and (b) voltage-capacity curves for the cells using various electrolytes; LiFSA/TEFP + VC (green), LiFSA/PNMePh + PS (red), and LiFSA/PNPrPr + VC (blue).

As shown in Fig. 1a, two-step Li stripping was observed in the super-concentrated electrolyte. The potential for the first step Li stripping was below zero volts *versus* Li^+^/Li. It was confirmed that this unique behavior was correct by CV measurements. The electrical capacity of the first step was 0.04 mA h cm^−2^. In contrast, the second step showed a large capacity, which indicates that this was the regular Li stripping. To understand the first step for Li stripping, we first made a Li plating/stripping test for the cell using electrolyte without VC as an additive. Fig. 3a shows cell voltage–capacity curves during Li stripping for cells using electrolytes with and without VC. The two-step Li stripping was not observed in the electrolyte without VC. The passivation film associated with PNMePh and VC will be prepared on Cu powder and Cu foil in our system. To cut effects of the two, the Li plating/stripping test was also conducted using only Cu foil as the electrode (Fig. S7†). The unit on the horizontal axis in Fig. S6† is electrical capacity per area of Cu foil. The cell with the Cu foil electrode showed the first step of Li stripping near zero volts *versus* Li^+^/Li. The electrical capacity per area of electrode was 0.02 mA h cm^−2^, and it was half of that for the cell with the Cu powder electrode.

To discuss the necessity of VC further, three kinds of super-concentrated electrolytes have been prepared, *i.e.*, LiFSA/TFEP + VC, LiFSA/PNMePh + PS, and LiFSA/PNPrPr. TFEP, PS, and PNPrPr are tris(trifluoroethyl) phosphate, 1,3-propanesultone, and dipropylamino-di(trifluoroethyl) phosphate, respectively (see Fig. S1†). We first have designed a super-concentrated electrolyte using TFEP as solvent instead of PNMePh. TFEP is one fluorinated alkyl phosphate with the self-extinguishing property. Unfortunately, the electrochemical cell using TFEP electrolyte exhibited no two-step stripping (Fig. 3b). The red line in Fig. 3b represents Li plating–stripping curve for the cell with (PS) as an electrolyte additive in place of VC. When Li stripping started, the cell voltage jumped immediately from −0.5 V to +0.26 V. The cell using LiFSA/PNPrPr + VC showed ambiguous 1^st^-step stripping. Thus, the two-step Li stripping will be associated with the super-concentration of LiFSA and PNMePh or PNPrPr involving VC. Therefore, the first step of Li stripping is associated with the thin film formed by the decomposition of VC and PN solvent. A possible chemical structure for the passivation film by the decomposition is shown in Fig. S8.†

### Characterization of Li electrodeposited on the Cu electrode

**Fig. 4:**
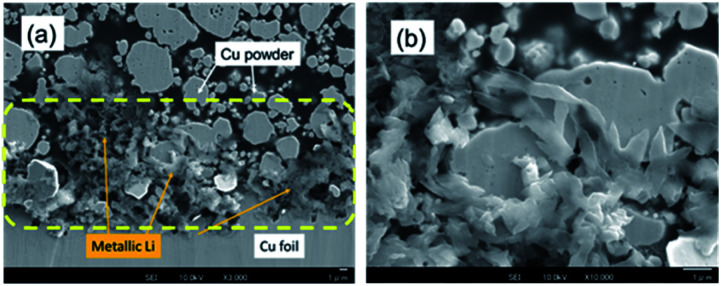
FE-SEM images of the Cu powder electrode cross-section after Li plating at magnifications of (a) 3000× and (b) 10 000×. The scale bar was 1 μm.

Li electrodeposition on the Cu powder electrode after the Li plating test to 0.78 mA h cm^−2^ was visualized using FE-SEM. Fig. 4a shows an FE-SEM image of the cross section of the Cu powder electrode after Li plating. The microscope magnification was 2000×. Many needle-like crystals were observed near the Cu foil (yellow dotted ellipses). An enlarged FE-SEM image of a needle-like crystal is shown in Fig. 4b. ^7^Li-NMR measurements revealed a strong peak at 260 ppm (Fig. S9†). Therefore, the needle-like crystals are attributed to metallic Li.^5^ The SEI films on the electrodeposited Li, *i.e.*, Li compounds,^32,33^ were further characterized by EDX analysis. We focused on some domains for a square area of less than 1 μm on one side and recorded the elemental distributions for C, O, F, and Cu. The EDX measurement positions on the sample after Li plating are shown in Fig. S10,† and the EDX results are summarized in Table S2.† The A to F analysis points correspond to SEIs on the electrodeposited Li. They contained 0.1–0.3 atom% of fluorine. The O/C ratio for the sample varied between 0.3 and 1.7. The SEI domains were divided into two ranges of 0.3 ≤ O/C ≤ 0.8 and 1.4 ≤ O/C ≤ 1.7, which indicate that two types of SEIs were present. The analysis points of G to K gave information regarding the thin films on the Cu powder. The films contained no fluorine, and the O/C ratio for the sample varied between 0.04 and 0.15.

### EIS analysis

**Fig. 5:**
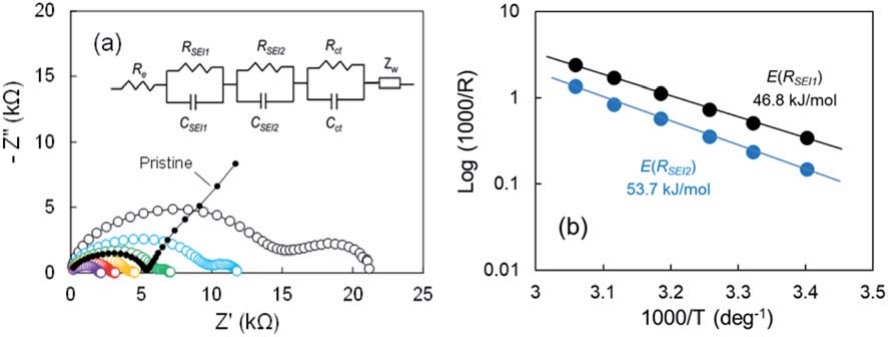
(a) Nyquist plots of the cell after the 2^nd^ Li plating for various temperatures; 21 °C (black), 28 °C (blue), 34 °C (green), 41 °C (orange), 48 °C (red), and 54 °C (purple), and (b) Arrhenius plots of 1/*R*.

According to our FE-SEM observation and EDX analysis, the SEI domains on the electrodeposited Li in Cu electrode were divided into two ranges of 0.3 ≤ O/C ≤ 0.8 and 1.4 ≤ O/C ≤ 1.7, which indicates that the two passivation films were present. To elucidate the effect of the two-type SEIs on Li stripping, we measured the impedance of the cell after the voltage relaxation adequately at various temperatures. EIS is a powerful method to obtain information on the electrochemical behaviour at Cu electrode–electrolyte interfaces. For the pristine sample before Li plating, one distorted semicircle and a linear portion of *Z*′ *vs. Z*′′ were observed. The Nyquist plot changed significantly as Li was plated. The second distorted semicircle was observed in the frequency range from 667 Hz to 94 mHz. The linear portion of *Z*′ *vs. Z*′′ appeared slightly between 94 mHz and 20 mHz. The impedance result is summarized by a relationship between reciprocals of the SEI resistance and the temperature to obtain the activation free energy. The Nyquist plots from 21 °C to 54 °C are shown in Fig. 5a. The 2^nd^ semicircle at the frequency of 667 Hz to 94 mHz faded as the temperature was increased. Fitting using the equivalent circuit in Fig. 5a was made to obtain *R*_SEI1_, *R*_SEI2_, and *R*_ct_. The activation free energy was calculated by using Arrhenius eqn (1),

1 *σ*(1/*R*_*n*_, *n* = 2, 3, or 4) = −Δ*E*_a_/*RT*

where *σ* is the conductivity, *E*_a_, is the activation free energy, *R* is Boltzmann's constant, and *T* is the temperature. The Arrhenius plots for the two SEIs for the semicircle are displayed in Fig. 5b. The activation free energies associated with Li transport through the SEI for *R*_SEI1_ and *R*_SEI2_ were 46.8 kJ mol^−1^ and 53.7 kJ mol^−1^, respectively. The activation free energies for the charge transfer *R*_ct_ was 49.5 kJ mol^−1^ (Fig. S11a†). *C*_SEI1_, *C*_SEI2_, and *C*_ct_ are described in Fig. S11b.† These results suggest a difference for the energy barrier associated with the SEI.

**Fig. 6:**
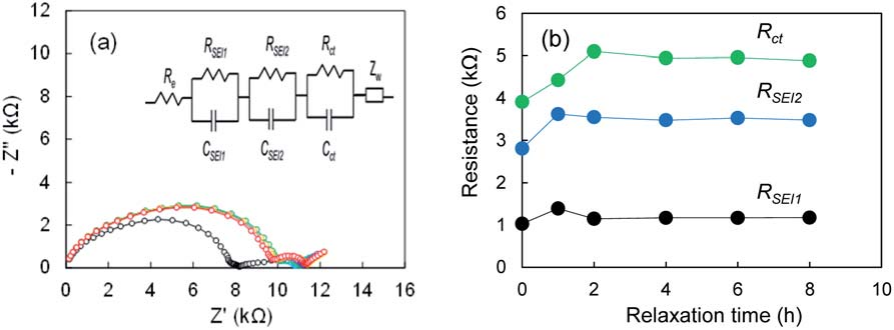
(a) Nyquist plots of the Li|Li cell after the cell fabrication, and (b) *R*_SEI1_, *R*_SEI2_, *R*_ct_; 0 h (black), 1 h (blue), 2 h (green), 4 h (purple), 6 h (orange), and 8 h (red). The electrolyte was LiFSA/PNMePh + VC.

To understand the formation of SEI on electrodeposited lithium further we measure Nyquist plots after cell fabrication for Li|Li cell using the super-concentrated electrolyte. When the electrolyte is touched with the electrodeposited Li, SEI forms immediately. In contrast, it may grow gradually in the super-concentrated electrolyte because of high viscosity of the electrolyte. As shown in Fig. 6a, the Nyquist plot changed drastically within one hour. The results suggest that the SEI formation finished within at least a few hours. It was concluded that the 1^st^-step stripping reflects the elimination of Li ions through the SEI in a transition period.

### Open-circuit voltage relaxation before the 1^st^-step Li stripping behaviour

**Fig. 7:**
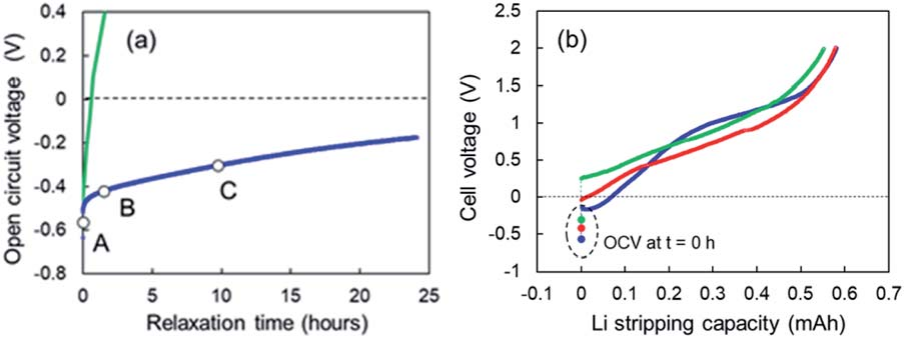
(a) OCV relaxation of the cells using the super-concentrated electrolytes with VC after Li plating of 0.35 mA cm^−2^ (green) and 0.78 mA cm^−2^ (blue), and (b) cell voltage–capacity curves during Li-stripping that started at the OCV of A (light blue), B (red) and C (green) shown in (a).

When Li plating stopped at 0.78 mA cm^−2^, slow open-circuit voltage (OCV) relaxation was observed. We examined the effect of the voltage relaxation on the first step of Li stripping. OCV monitoring is a practical *in situ* Li plating detection method to examine the onset of Li plating and fast charging on a graphite anode in conventional Li-ion batteries.^34,35^ Uhlmann *et al.* first reported a characteristic OCV profile after charging; two potential plateaus for the plated Li and lithiated graphite (Li_*x*_C_6_).^36^ Schindler *et al.* proposed a Li detection technique using the potential decay curve as a voltage derivative.^37^ Data for these OCV-based techniques could be easily collected in the 10 min immediately after charging. To understand the two-step Li stripping in this study, we have monitored OCV relaxation immediately after Li plating. The cells using Cu powder after Li plating of 0.35 mA cm^−2^ and 0.78 mA cm^−2^ was tested. The former sample shows voltage relaxation at relatively high speed (green line in Fig. 7a). The OCV reached 0 V within thirty minutes. The OCV reached over +1.5 V at 5 h, indicating no electrodeposited Li in Cu electrode. In contrast, the OCV in the latter relaxed slowly with an order of dozens of hours (blue line in Fig. 7a). It came to −0.2 V after 24 h. This result suggests a possibility of existence of Li ions that are difficult to be electrodeposited. Next, Li stripping tests were conducted at various OCV points to find out the impact of slow voltage relaxation. The cell voltage–capacity curve moved to the positive side with the increase of OCV (Fig. 7b). Although the first step region of Li stripping was indistinctive, the capacities for the Li stripping were almost the same. According to these results, we can see that the SEI formation has completed during the relaxation.

**Fig. 8:**
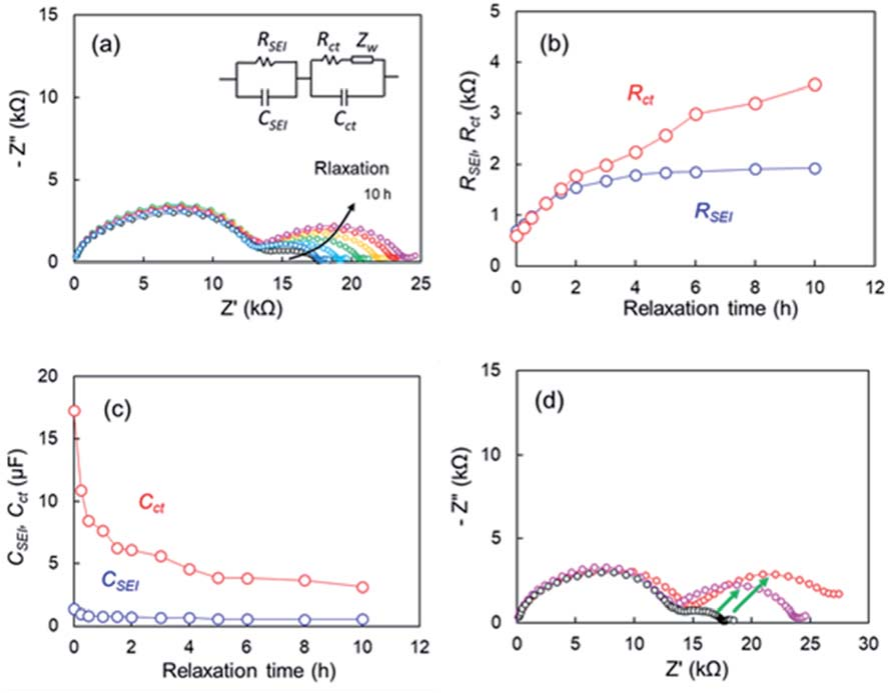
(a) Nyquist plots during voltage relaxation after Li plating of the cell using the super-concentrated electrolytes with VC, (b) resistances (c) capacitances during voltage relaxation, (d) comparison of Nyquist plots after voltage relaxation and electrochemical Li stripping after relaxation time, *t* = 0 h (black), *t* = 10 h (pink), and after Li stripping up to +2 V (red).

EIS measurements corresponding to points after various relaxation times *t*, were conducted to determine the effect of the OCV relaxation on the 1^st^ step stripping. The Nyquist plots appeared to vary over relaxation time (Fig. 8a). The black and pink lines represent the Nyquist plots corresponding to point of *t* = 0 h and *t* = 10 h, respectively. When the OCV relaxation proceeded, the second distorted semicircle enlarged drastically. An equivalent circuit composed of *R* and two parallel units of resistance and capacity (*R*_SEI_|*C*_SEI_, *R*_ct_|*C*_ct_), as shown in Fig. 8a, was used to analyze the impedance spectrum for the second semicircle, where *R*_SEI_ and *C*_SEI_ are the resistance and capacitance^38,39^ of the SEI on the metallic Li deposited, respectively, and *R*_ct_ and *C*_ct_ are characteristic for the charge-transfer of lithium metal-SEI and lithium metal–electrolyte interfaces at low frequencies.^40^ A one-time EIS measurement time was *ca.* 10 min; therefore, the EIS is a useful *in situ* technique for the voltage relaxation behaviour. Fig. 8b shows *R*_SEI_ and *R*_ct_ during the relaxation. The capacities for *C*_SEI_ and *C*_ct_ are displayed in Fig. 8c. A significant increase in *R*_ct_ (red circle) is the most distinctive among the two resistances, and it reached four times of the initial value. For the capacity elements, a drastic decrease in *C*_ct_ is remarkable. It was therefore concluded that the voltage relaxation was associated with the change in *C*_ct_. Fig. 8d compares Nyquist plots for the cells after voltage relaxation (*t* = 10 h) and after electrochemical charge up to +2 V. The black symbols in Fig. 8d represent Nyquist plot for the cell before relaxation or charge. The growth in the second semicircle was noticeable for the plots in the both specimens after relaxation and charge (see green arrows). These results suggest that Li ions disappeared from the Cu electrode in the voltage relaxation. Here we take a quantitative study for OCV relaxation. The capacitance for the charge-transfer *C*_ct_ was about 10 μF, and the cell voltage for the 1^st^-step stripping was approximately −0.1 V. Therefore, the electrical capacity was calculated to be 2.78 × 10^−5^ mA h (=0.278 mA h × 10^−5^ F/0.1 V). In contrast, the electrical capacity observed for the 1^st^-step stripping was 0.05 mA h. The results indicate that the charge-transfer capacitance of Li ions for which it is difficult to be electrodeposited was insignificant contribution to the 1^st^-step stripping. It was thus concluded that the electrical capacitance for the slow relaxation was not main factor in the 1^st^-step stripping. The cell using only Cu foil also showed similar relaxation behaviour. The EIS data for the cell are presented (Fig. S12†).

**Fig. 9:**
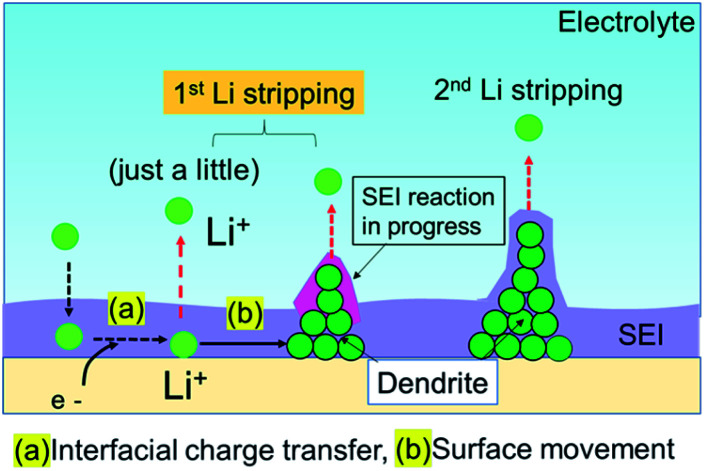
Schematic illustration of the Li stripping process.

The Li plating process is explained by multi-step reaction; first, mass transfer of Li^+^ in the electrolyte and subsequent mass transfer of Li^+^ in the SEI occurs. The Li ion receives an electron at the surface of the Cu electrode during Li plating and subsequently forms a charge-transfer state. Finally, growth of a Li nucleus and formation of a Li dendrite arise *via* surface movement processes and nanoscale nucleation.^41^ The mass transfer current is linear to diffusion coefficient *D* of Li ion, which *D* is related to viscosity of electrolyte *η* by the Stokes–Einstein equation, *D* = *kT*/6π*rη*.^42,43^ Since the viscosity of the super-concentrated in this study was 351 mPa s (Fig. S13†), the mass transfer occurs slowly in the super-concentrated electrolyte. The *in operando* EIS measurements for voltage relaxation suggest that Li^+^ ions that are difficult to electrodeposit are present in Cu electrode immediately after Li plating or the possibility of quasi-stable stated Li ions (Fig. S14†). Those Li^+^ ions have a long lifetime on the order of tens of hours and are associated with the interface charge transfer. Therefore, they are easily removed from the Cu electrode qualitatively in comparison with those from electrodeposited metallic Li. They exhibit lower potential than that for the regular dissolution of electrodeposited Li. Fig. 9 shows a schematic illustration for the Li stripping behaviour. When Li ions migrate in the SEI film and reach at the surface of Cu electrode, Li electrodeposition starts. As shown in Fig. 4, many dendritic Li, not spherical Li, grow during Li plating. This is because the current density is too small (0.033 mA cm^−2^).^44^ The EDX and EIS analysis results indicated two kinds of SEIs with different composition with different activation free energies associated with Li transport through the SEI. One of the two SEIs (pink in Fig. 9) was in the transition state of formation, and exhibited lower potential than that for the regular dissociation of electrodeposited Li. The 1^st^-step stripping contained a small amount of capacity for Li ions that were difficult to electrodeposit or steady state Li ions.

## Conclusions

When the electrode potential goes below the standard Li^+^/Li plating level, Li^+^ ions in the electrolyte are theoretically electrodeposited on the electrode. In this study, Li plating tests on Cu powder electrodes were conducted in a super-concentrated electrolyte of LiFSA and PNMePh with VC. Two-step Li stripping was observed; the potential plateaus for the 1^st^ and 2^nd^ stripping steps appeared at −0.2 V and at +1.0 V *vs.* the Li metal counter electrode, respectively. They were caused mainly by different passivation layers due to the electrolyte reduction reaction, which were detected at the surfaces of electrodeposited Li in Cu electrode. The former passivation layer was in a transition period of the SEI formation. The 1^st^-step stripping contained the stripping by Li ions for which it is difficult to electrodeposit or a quasi-stable state of Li^+^ ions which is formed during the Li plating process. This unique behaviour was observed at a large overpotential induced by the high viscosity of electrolyte, which also facilitated the development of the SEI, which was formed by the decomposition of PNMePh and VC. This unexpected discovery stemmed from investigation of the Li plating/stripping under low current mode in the super-concentrated electrolyte.

## Conflicts of interest

The authors declare no competing financial interest.

## Supplementary Material

RA-011-D1RA01490K-s001
