# Supplementary material for: Observation of lithium stripping in super-concentrated electrolyte at potentials lower than regular Li stripping
Source: RSC Adv. 2021 Apr 9;11(22):13359–65. doi: 10.1039/d1ra01490k (PMC8697623; doi:10.1039/d1ra01490k)
Supplement: RA-011-D1RA01490K-s001 [file RA-011-D1RA01490K-s001.pdf]

Electronic Supplementary Material (ESI) for RSC Advances.
This journal is © The Royal Society of Chemistry 2021

Supplementary information

Observation of lithium stripping in super-concentrated electrolyte at potentials lower than regular Li stripping

Tohru Shiga, Yumi Masuoka, and Hiroshi Nozakio*

Toyota Central Research & Development Laboratories Inc.
Yokomichi, Nagakute-city, Aichi-ken, 480-1192 Japan
*E-mail: e0560@mosk.tytlabs.co.jp

1. Materials

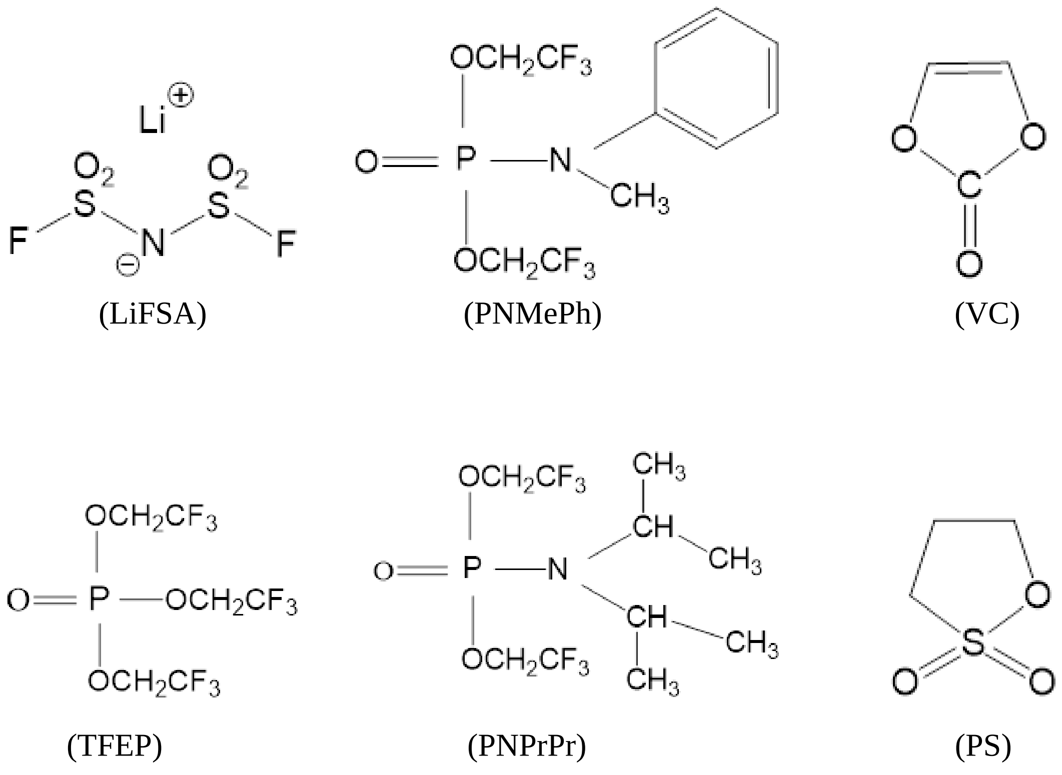

Figure S1. Chemical structures of materials.

2. Experimental

2.1. Electrode

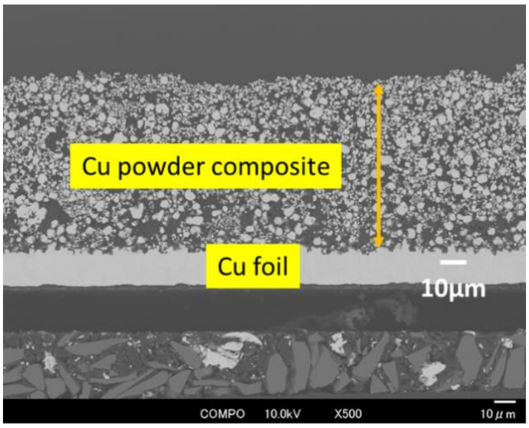

Figure S2. FE-SEM image of the cross section of a Cu powder electrode.

2.2. Electrochemical cell

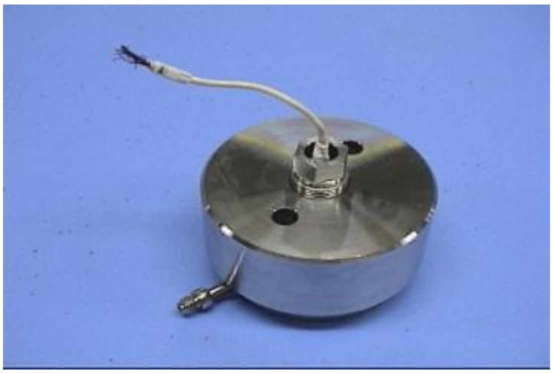

Figure S3. Photograph of an electrochemical cell.

Table S1. ^{7}Li-NMR measurement parameters

Measurement frequency	155.5080156 MHz
Spectral width	100 kHz
Pulse width	1.0 μm (30°pulse)
Pulse repetition time	12 s
Observation point	8192 points
Reference material	1M LiCl aquous solution
Temperature	Room temperature
Rotational frequency	0 Hz
Sample tube property	ZrO_2, inner diameter 1 mm<br>Length 2.5 mm

3. Results

3.1. Li plating behavior for the cell fabricated using only Cu powder

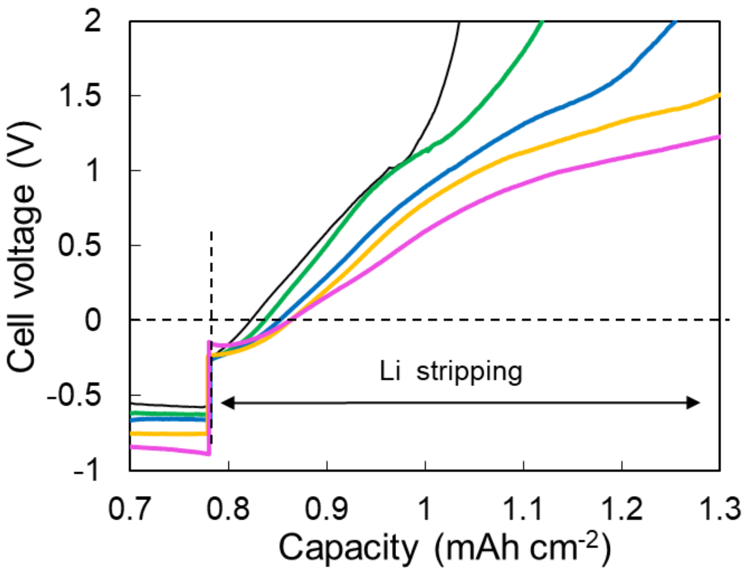

Figure S4. Cu electrode potential-capacity curve for the electrode fabricated using only Cu powder during the initial stage of Li stripping.

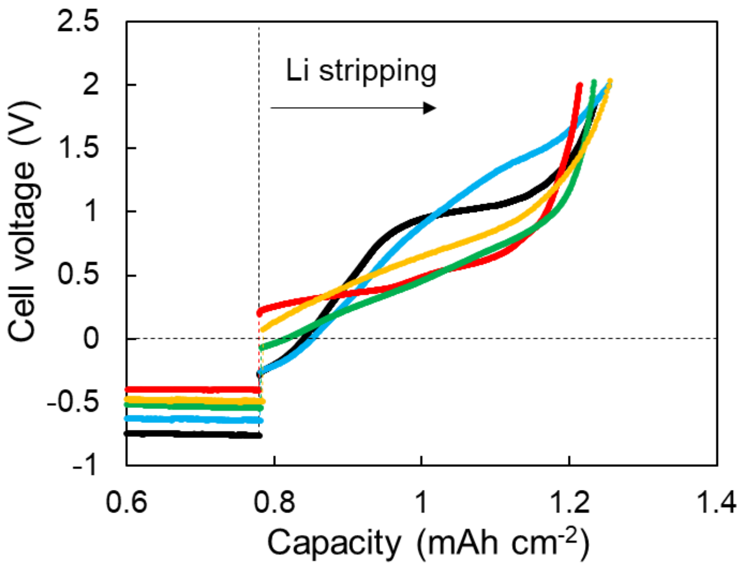

Figure S5. Cell voltage-capacity curves for the cells using various LiFSA concentrations during the initial stage of Li stripping; [LiFSA]/[PNMePh] =0.125 (red), 0.2 (orange), 0.25 (green), 0.33 (blue), and 0.4 (black).

.

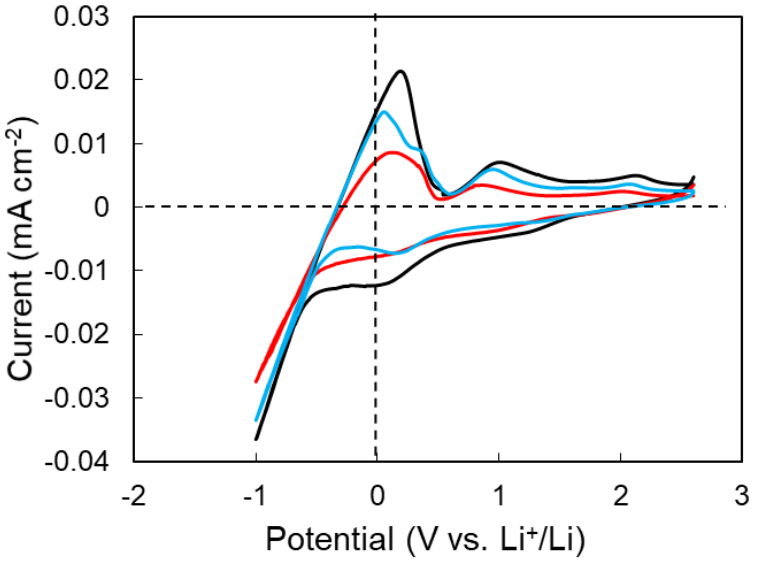

Figure S6. CV curves for the cell using Cu powder electrode under various sweep rates; 0.1 mV s^{-1} (red), 0.2 mV s^{-1} (blue), and 0.5 mV s^{-1} (black).

3.4. Li plating/stripping test in some electrolytes

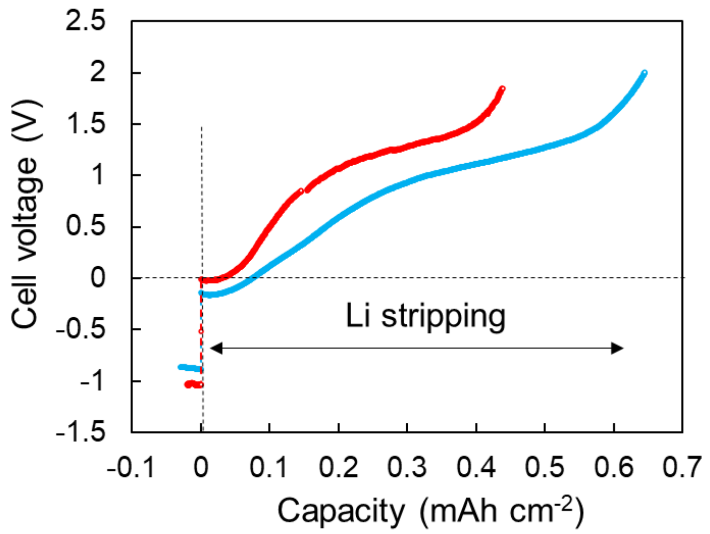

Figure S7. 3rd cycle voltage-capacity curves for the cells using the super-concentrated electrolytes with powder (blue) and only Cu foil (red) VC during the Li stripping process.

3.5. Chemical structure of SEI associated with VC

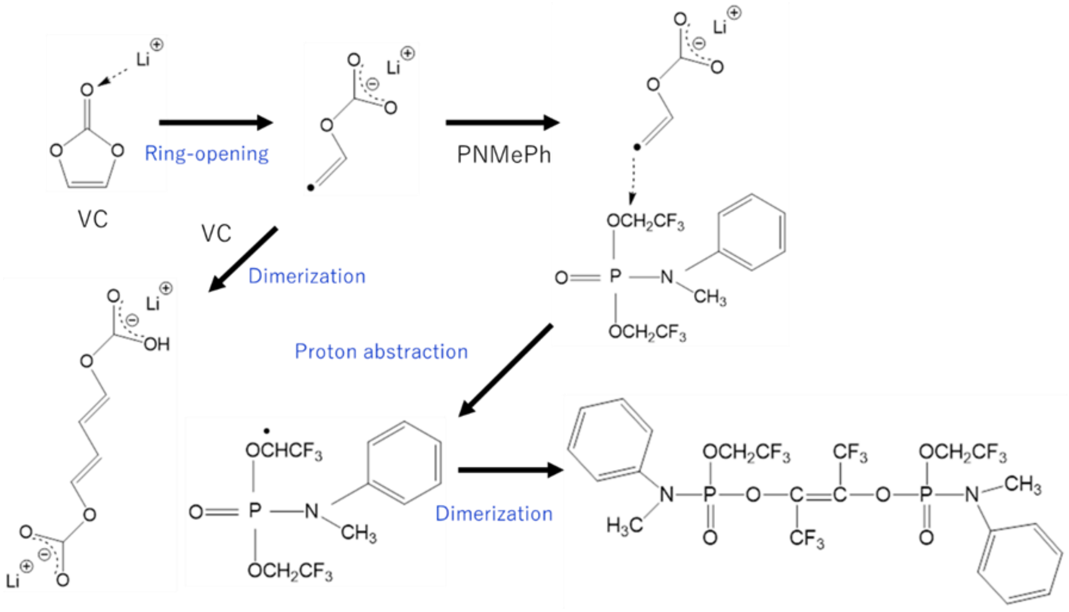

Figure S8. Reaction scheme for SEI formation.

3.6. ^{7}Li-NMR results

The broad signal at approximately 0 ppm provided information on the Li^+ ions in LiFSA and Li compounds that result from the reductive decomposition of the electrolyte by metallic Li. Waveform deconvolution was performed for the broad NMR peak at ca. 0 ppm. The NMR waveform was deconvoluted into three waves with peaks at −0.63 ppm, −1.32 ppm, and 3.25 ppm, the percentages of which were 32%, 14%, and 54%, respectively. The wave with the peak at −1.32 ppm is attributed to Li^+ ions in the LiFSA/PNMePh electrolyte because it was observed in the spectrum of the pristine sample before the Li plating test. The other two waves reflected reductive decomposition. Letellier et al.[1] studied the SEI with a $LiPF_6$/(EC+DMC) electrolyte using ^{7}Li-NMR and identified Li_2CO_3, LiF, Li_2O, LiOH, and $ROCO_2Li$ as SEI components. The ^{7}Li-NMR signal of LiF appears between 0 and −1 ppm; therefore, the wave with the peak at −0.63 ppm in this study may be due to an analogue of LiF. The ^{7}Li-NMR signal of Li_2CO_3 was detected at 3.89 ppm; therefore, the Li compound with a peak at 3.25 ppm is most likely an oxide.

(1) Letellier, M.; Chevallier, F.; Morcrette, M. In Situ ^{7}Li-Nuclear Magnetic Resonance Observation of the Electrochemical Intercalation of Lithium in Graphite; 1st Cycle. *Carbon,* **2007,** *45,* 1025-1034.

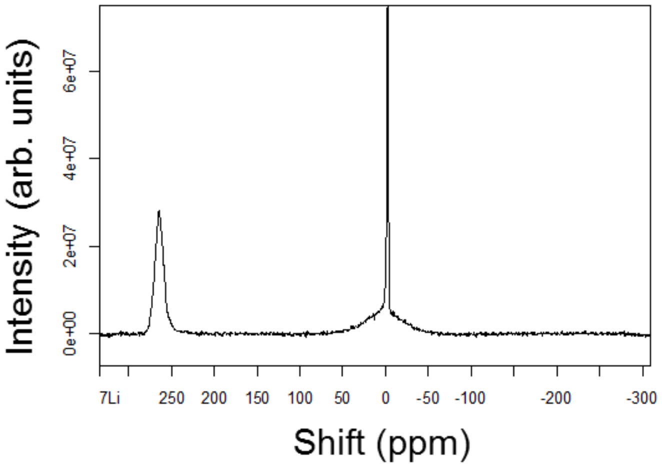

Figure S9. ^{7}Li-NMR spectrum for a Cu powder electrode after Li plating.

3.7. EDX results

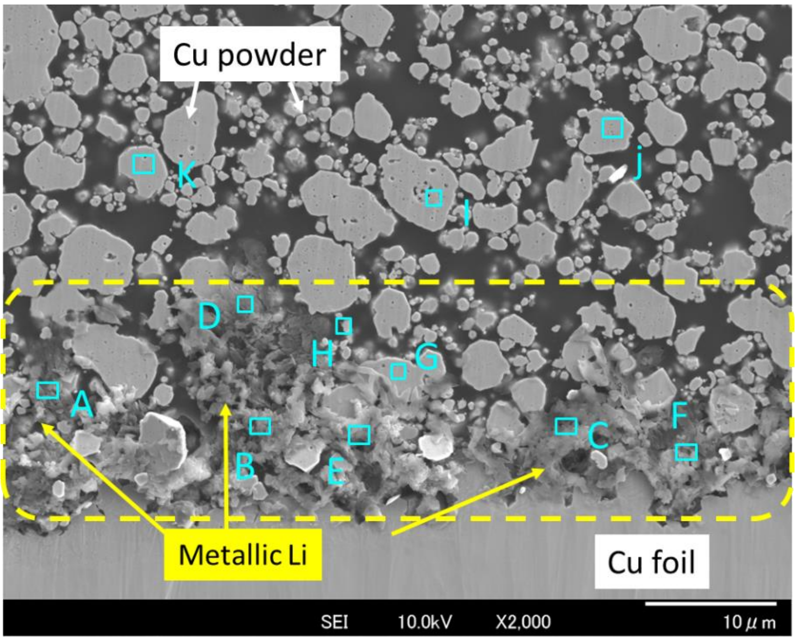

Figure S10. EDX measurement points on the Cu powder electrode after Li plating.

Table S2. EDX results for the Cu powder electrode after Li plating

No.	C	O	F	Cu	O/C
A	39.8	55.8	0.1	4.3	1.41
B	75.9	29.5	0.3	4.4	0.39
C	54.2	26.9	0.2	18.7	0.49
D	35.8	60.5	0.1	3.6	1.69
E	42.4	34.1	0.3	23.1	0.81
F	40.3	52	0.2	7.5	1.29
G	12.6	0.9	0	86.3	0.071
H	24.6	0.9	0	74.5	0.037
I	11.2	0.2	0	88.6	0.018
J	13.1	1.6	0	85.3	0.122
K	13.5	2	0	84.6	0.148

3.8. EIS results for the cell using Cu powder

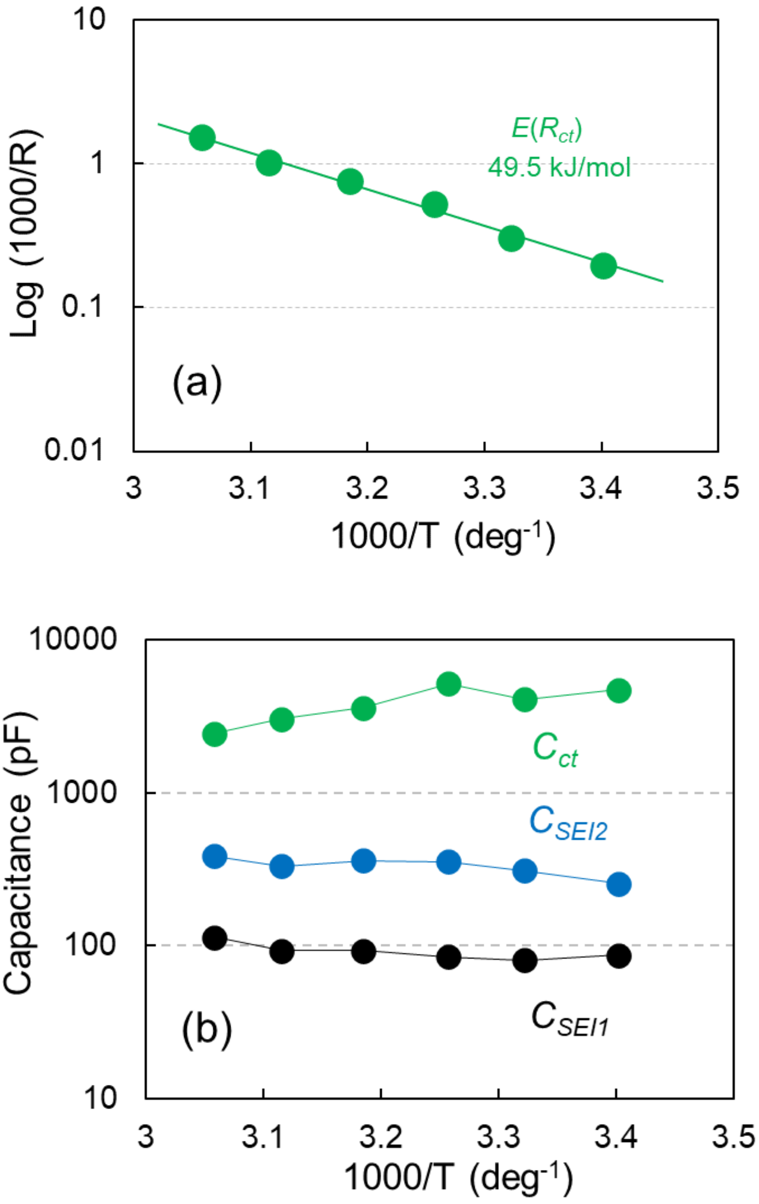

Figure S11. (a) Arrhenius plots of $1/R_{ct}$, and (b) C_{SEI1}, C_{SEI2}, and C_{ct}

3.9. Relaxation and EIS results for the cell using only Cu foil

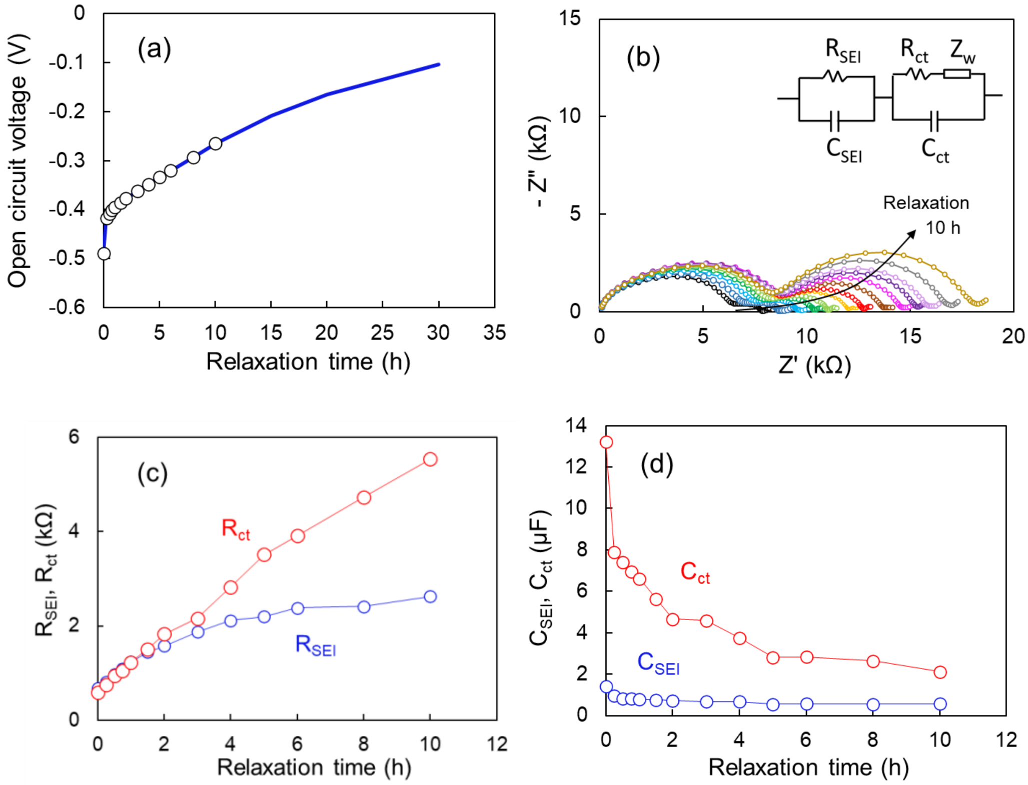

Figure S12. (a) OCV relaxation of the cells using the super-concentrated electrolytes with VC after Li plating, and (b) Nyquist plots, (c) Resistance factors, and (d) Electrical capacities during OCV relaxation; relaxation time from t=0 h to 10 h,

3.10. Viscosity of the electrolyte

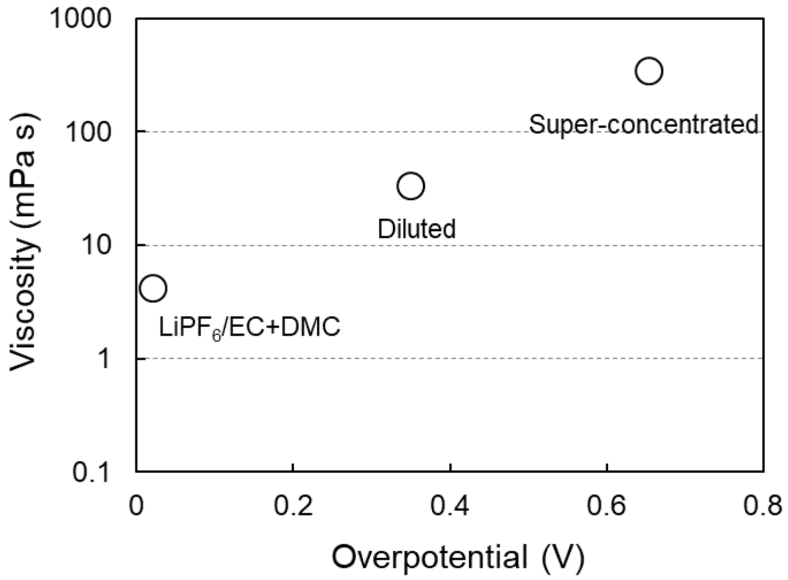

Figure S13. Relationship between viscosity of the electrolyte and overpotential during Li plating.

3.11. Energy diagram for Li deposition

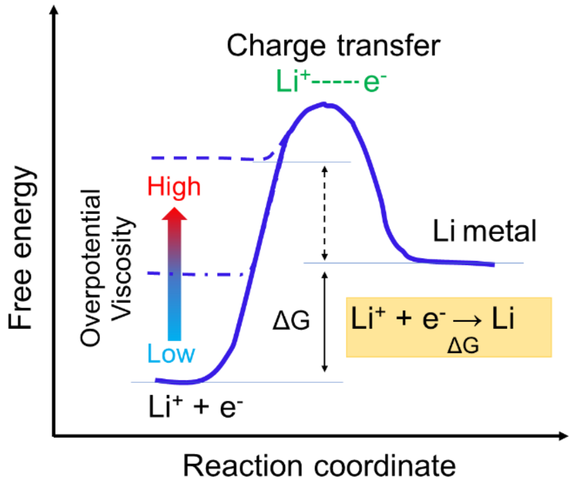

Figure S14. Energy diagram during Li plating: Relationship between viscosity of the electrolyte and overpotential.
